# Deep learning for the diagnosis of mesial temporal lobe epilepsy

**DOI:** 10.1371/journal.pone.0282082

**Published:** 2023-02-23

**Authors:** Kyoya Sakashita, Yukinori Akiyama, Tsukasa Hirano, Ayaka Sasagawa, Masayasu Arihara, Tomoyoshi Kuribara, Satoko Ochi, Rei Enatsu, Takeshi Mikami, Nobuhiro Mikuni

**Affiliations:** Department of Neurosurgery, Sapporo Medical University, Sapporo, Japan; Nanyang Technological University, SINGAPORE

## Abstract

**Objective:**

This study aimed to enable the automatic detection of the hippocampus and diagnose mesial temporal lobe epilepsy (MTLE) with the hippocampus as the epileptogenic area using artificial intelligence (AI). We compared the diagnostic accuracies of AI and neurosurgical physicians for MTLE with the hippocampus as the epileptogenic area.

**Method:**

In this study, we used an AI program to diagnose MTLE. The image sets were processed using a code written in Python 3.7.4. and analyzed using Open Computer Vision 4.5.1. The deep learning model, which was a fine-tuned VGG16 model, consisted of several layers. The diagnostic accuracies of AI and board-certified neurosurgeons were compared.

**Results:**

AI detected the hippocampi automatically and diagnosed MTLE with the hippocampus as the epileptogenic area on both T2-weighted imaging (T2WI) and fluid-attenuated inversion recovery (FLAIR) images. The diagnostic accuracies of AI based on T2WI and FLAIR data were 99% and 89%, respectively, and those of neurosurgeons based on T2WI and FLAIR data were 94% and 95%, respectively. The diagnostic accuracy of AI was statistically higher than that of board-certified neurosurgeons based on T2WI data (p = 0.00129).

**Conclusion:**

The deep learning-based AI program is highly accurate and can diagnose MTLE better than some board-certified neurosurgeons. AI can maintain a certain level of output accuracy and can be a reliable assistant to doctors.

## 1. Introduction

Temporal lobe epilepsies (TLEs) are classified into three types based on the region of occurrence: the limbic system, mesial TLE (MTLE); neocortex, lateral TLE; and mixed TLE. MTLE involves parts of the limbic system (such as the amygdala and hippocampus) and is thought to be an independent epileptic syndrome associated with unique epileptic seizures and different treatment outcomes. In 1954, Penfield proposed that MTLE is part of a disease group caused by hippocampal sclerosis (HS) [1]. The epileptogenic region in MTLE is located within the limbic system (the hippocampus, amygdaloid body, uncus, and parahippocampal gyrus) of the temporal lobe, whereas that for lateral TLE is located on the lateral surface of the temporal lobe neocortex.

Patients with MTLE experience some distinct symptoms, such as cacosmia, flashback, uneasiness, a sense of fear, and compound visual and auditory hallucinations, perceived as aura. After aural sensation, symptoms of epileptic seizure, such as staring, arrest of movement, and various types of automatisms (such as fumbling for clothes, cheek-biting, and fidgeting), may occur. Lightheadedness persists for 5 to 10 minutes after attack symptoms. The signs of an attack in lateral TLE are acousma (simple sounds such as ringing, but not words) and dizziness.

The most common cause of TLE is HS. HS is diagnosed based on the presence of unilateral hippocampal atrophy and hyperintensities on the T2-weighted magnetic resonance imaging (MRI) or fluid-attenuated inversion recovery (FLAIR) images. In addition, HS is rarely observed in both hippocampi. Seizure control with antiepileptic drug treatment is possible in 70%–80% of patients; however, 20%–30% of patients have drug-resistant intractable epilepsy [2]. Therefore, approximately 10% of all epileptic patients require surgical treatment.

The surgical treatment of TLE results in an 80% reduction in epileptic seizures; however, surgical treatment is less effective against other types of epilepsy. Surgical methods such as anterior temporal lobectomy (ATL) and selective amygdalohippocampectomy are used for treating TLE. Moreover, the surgical resection of mesial structures (hippocampus and amygdala) was first described in the 1950s [3]. Although removal of the lateral temporal cortex was performed during earlier times, the selective removal of the epileptogenic lesion has only recently become mainstream. In ATL, approximately 35–45 mm of tissue from the tip of the temporal lobe cortex toward the inferior horn of the lateral ventricle is removed, and thereafter, the hippocampus and amygdala are resected. ATL provides a relatively wide surgical field for observing the structures. However, this method has certain limitations. Complications such as memory disturbances and upper-quarter homonymous hemianopsia commonly occur after ATL. Moreover, resection on the dominant side may lead to the impairment of the language center located at the tip of the temporal lobe [3]. Hence, neurosurgeons often hesitate to further resect the lateral regions of the temporal lobe cortex.

Furthermore, diagnosing HS is crucial as the hippocampus is important for cognitive functions such as memory and spatial discrimination [4]. In addition, diagnosis of HS can be difficult, even for neurologists or neurosurgeons [5–7]. Neuropathological conditions of HS are mainly characterized by hippocampal neuronal loss. In 2013, the International League Against Epilepsy proposed a classification system for identification of pathological subtypes of HS based on the patterns of neuronal loss [8]. Atrophy and/or high signal intensity of hippocampi on T2-weighted imaging (T2WI) and FLAIR are the most reliable findings of HS. These findings reflect the neuropathological features of HS [9].

In Japan, diagnosis based on neuroimaging is usually performed by neurosurgeons. The 2018 report of Labor Standards Bureau, Ministry of Health, Labor and Welfare, revealed that the number of radiologists in Japan was 6,813, whereas the number of doctors in Japan was 311,963. Therefore, Japanese doctors should be able to reach a diagnosis using neuroimages by themselves.

In this study, we present an alternative diagnosis method using artificial intelligence (AI), which involves automatic detection of the hippocampus and diagnosis of MTLE with the hippocampus as the epileptogenic area. Here, we demonstrated that the deep learning-based AI program successfully detects the hippocampus and therefore, epileptic attacks because of MTLE with the hippocampus as the epileptogenic area based on MRI, with an accuracy of > 90%.

## 2. Methods

### 2.1. Patients

The human and animal studies were approved by the Ethics Committee of Sapporo Medical University Hospital. The study was conducted in accordance with the ethical principles of the Declaration of Helsinki (1964) and its later amendments. The need for consent was waived by the ethics committee.

**Fig 1:**
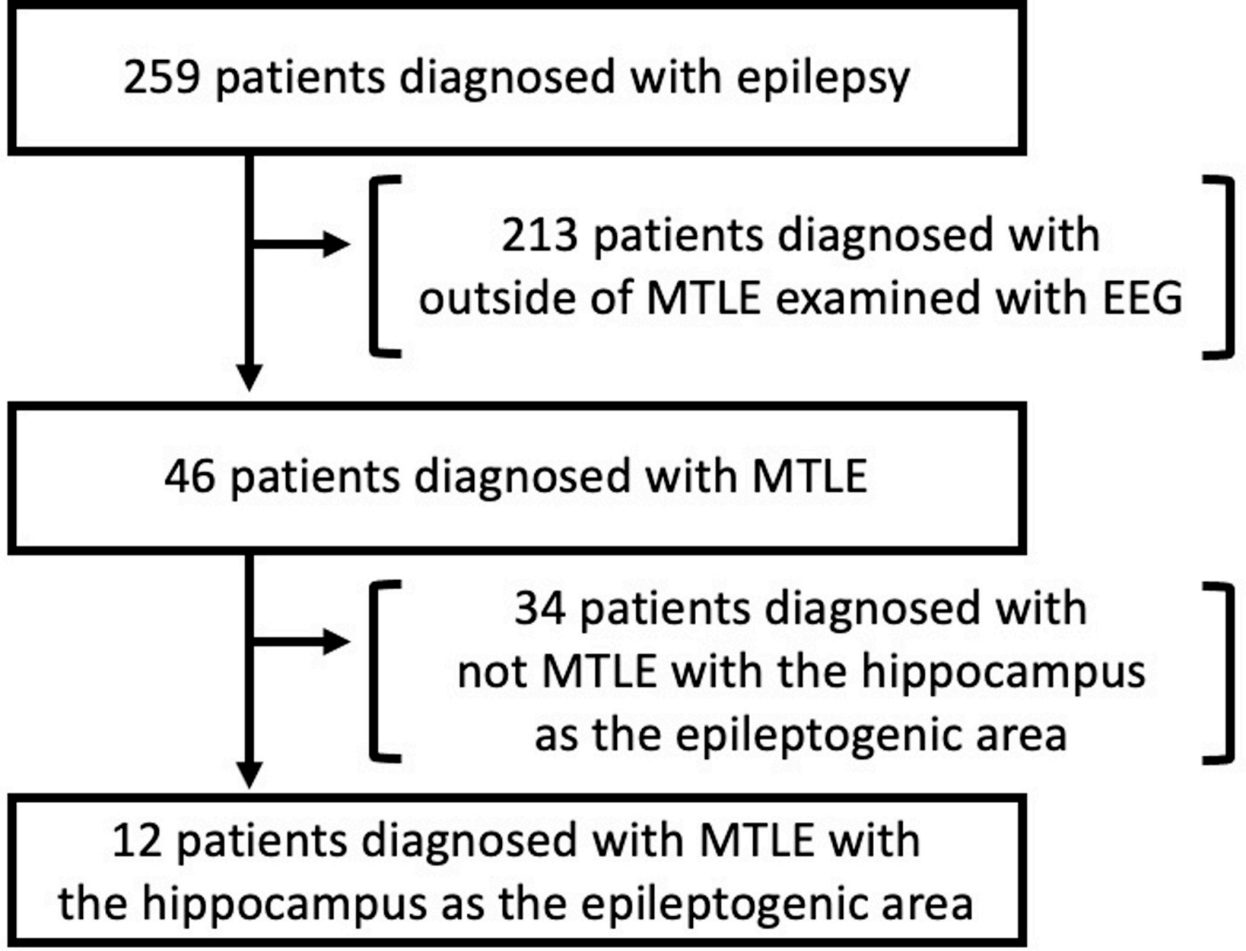
Flow chart for the diagnosis of MTLE with hippocampal epileptogenic area by artificial intelligence (AI) and board-certified neurosurgeons. The data of 259 patients with epilepsy were retrospectively analyzed. However, 213 patients without MTLE were excluded. Twelve patients with confirmed diagnosis of MTLE, with the epileptogenic area in the hippocampus, were enrolled in the study.

This study included all consecutive patients diagnosed with epilepsy at our hospital between January 2016 and December 2019 who had not been previously treated. A total of 259 patients (128 men and 131 women) were enrolled and analyzed retrospectively. Forty-six patients were diagnosed with MTLE, whereas 12 patients were diagnosed with MTLE with the hippocampus as the epileptogenic area (Fig 1). Patients diagnosed with MTLE with the hippocampus as the epileptogenic area were selected because they had hippocampal atrophy, hyperintensities on FLAIR MRI and spikes in the mesial temporal lobe on intracranial electroencephalography (EEG). Patients who were found to be seizure-free in a presurgical evaluation with subdural electrodes underwent resection surgeries at the Sapporo Medical University. Two or three continuous coronal sections of T2WI at the level of the anterior commissure from patients with MTLE and those with other diseases such as psychogenic nonepileptic seizures (control subjects) were prepared for AI analysis. MR images were allocated to two datasets: learning and test datasets. The learning data were used for deep learning, whereas the test data were used for analyzing the diagnostic accuracy (i.e., validation). The test data included 30% of all data. MRI data of patients with MTLE with the hippocampus as the epileptogenic area included four images on T2WI (25.0%) and six images on FLAIR (21.4%). MRI data of control subjects included 77 images on T2WI (36.5%) and 77 images on FLAIR (27.7%). After the deep learning process, the diagnostic accuracy was calculated using the test data from T2-weighted images and FLAIR images.

All data were fully anonymized before we accessed them.

### 2.2. MRI examination

The basis for pre-processing was T2WI of the head using a clinical 1.5- or 3.0-Tesla magnetic resonance scanner (Signa HDxt 3.0 Tversion16®, GE Healthcare, Connecticut, USA). The imaging parameters for the T2-weighted fast-spin echo imaging were as follows: flip angle, 90°; repetition time, 5000 ms; echo time, 102.0 ms; bandwidth, 50.0 kHz; field of view, 200 mm × 200 mm; scan thickness, 4.0 mm; slice gap, 1.0 mm; number of slices, 26–30; matrix, 352 × 256; number of signals averaged, 1; and imaging time, 1 min 05 s. The imaging parameters of FLAIR imaging were as follows: flip angle, 90°; time of repetition, 5000 ms; echo time, 102.0 ms; bandwidth, 50.0 kHz; field of view, 200 mm × 200 mm; scan thickness, 4.0 mm; slice gap, 1.0 mm; number of slices, 26–30; matrix, 352 × 256; number of signals averaged, 1, and imaging time = 1 min 05 s.

### 2.3. Data processing

We used an HP Z840 workstation (Hewlett-Packard Company, Palo Alto, California, USA) with a Core Xe-6700 K 4.00 GHz (Intel, Santa Clara, CA, USA) central processing unit, 64 GB of random access memory, and a GeForce GTX 1080 (NVIDIA, Santa Clara, CA, USA) graphics processing unit for the training phase of deep learning. The image sets were processed using a code written in Python 3.7.4 (http://www.python.org) and Pillow 3.3.1 (http://pypi.python.org/pypi/Pillow/3.3.1), which is a python imaging library. We used the Open Computer Vision (OpenCV) 4.5.1 (https://docs.opencv.org/4.5.1/) and Keras (version 2.3.0), a framework for neural networks and a part of the Tensorflow (version 1.14.0) platform. OpenCV is a library of programming functions that is free for use under an open-source BSD license (OpenCV. Open Source Computer Vision Library. 2015.). OpenCV was used for hippocampal detection. MRI images were obtained of 113 T2WI and 148 FLAIR cases with a diagnosis of epilepsy from January 2016 to December 2019. On both the left and right sides, 226 T2WI and 296 FLAIR images with a resolution of 96 × 96 pixels were used for hippocampal images, and 327 T2WI and 367 FLAIR images with a resolution of 96 × 96 pixels were used for non-hippocampal images after amplification using augmentation. Haar-like features and local binary patterns (LBPs) were used as features. Hyperparameters of the created cascade classifier, mainly scaleFactor, minNeighbors, and minSize, were adjusted for optimization. Image processing was performed separately for the training and test image sets to prevent overlearning during the training phase. Data augmentation was performed for the training data sets; thus, the number of images from patients with MTLE with the hippocampus as the epileptogenic area and from control subjects increased from 12 to 1134 and 134 to 7344 for T2WI, and 22 to 1975 and 201 to 8635 for FLAIR, respectively. In addition, data augmentation was performed for the validation data sets; thus, the number of images from patients with MTLE with the hippocampus as the epileptogenic area and from control subjects increased from 4 to 392 and 77 to 5359 for T2WI, and 6 to 580 and 77 to 5369 for the FLAIR images, respectively. After data augmentation, the total number of images in the training dataset for T2WI was 8478, and for FLAIR was 10610, and that in the validation dataset for T2WI was 5751 and for FLAIR was 5949 (Tables 1 and 2).

**Table 1:** Image processing.

	MTLE with the hippocampus as the epileptogenic area			control			total		
	training		test	training		test	training		test
	original	augmentation	original	original	augmentation	original	original	augmentation	original
T2WI	12	1134	4	134	7344	77	146	8478	81
FLAIR	22	1975	6	201	8635	77	223	10610	83

**Table 2:** Validation data set.

sex	age	laterality	Diagnosis	EEG findings (spikes regional)	symptoms, seizure type
M	9	left	MTLE with the hippocampus as the epileptogenic area	left temporal & spike and wave generalized	dialeptic seizure, oral automatism, automotor seizure
F	20	left	MTLE with the hippocampus as the epileptogenic area	left temporal	abdominal aura, dialeptic seizure, focal impaired awareness seizure
F	22	left	MTLE with the hippocampus as the epileptogenic area	left temporal	dialeptic seizure, GTCS
M	22	right	MTLE with the hippocampus as the epileptogenic area	right temporal	dialeptic seizure, automotor seizure
F	22	right	MTLE with the hippocampus as the epileptogenic area	right temporal	automotor seizure
M	30	right	MTLE with the hippocampus as the epileptogenic area	right front-temporal	dialeptic seizure
M	38	right	MTLE with the hippocampus as the epileptogenic area	right front-temporal	dialeptic seizure, GTCS
F	39	left	MTLE with the hippocampus as the epileptogenic area	left temporal	abdominal aura, aphasic
M	42	right	MTLE with the hippocampus as the epileptogenic area	right temporal	abdominal aura, dialeptic seizure, automotor seizure
F	45	right	MTLE with the hippocampus as the epileptogenic area	right and left temporal	abdominal aura, automotor seizure

MTLE, mesial temporal lobe epilepsy; GTCS, generalized tonic-clonic convulsion; EEG, electroencephalogram.

The image data generator created varied images using rotation, width and height shift, horizontal and vertical flip, zoom, and shear. This type of data augmentation is commonly used in deep learning (the code is available at https://keras.io/) for small datasets such as those of medical diseases.

**Fig 2:**
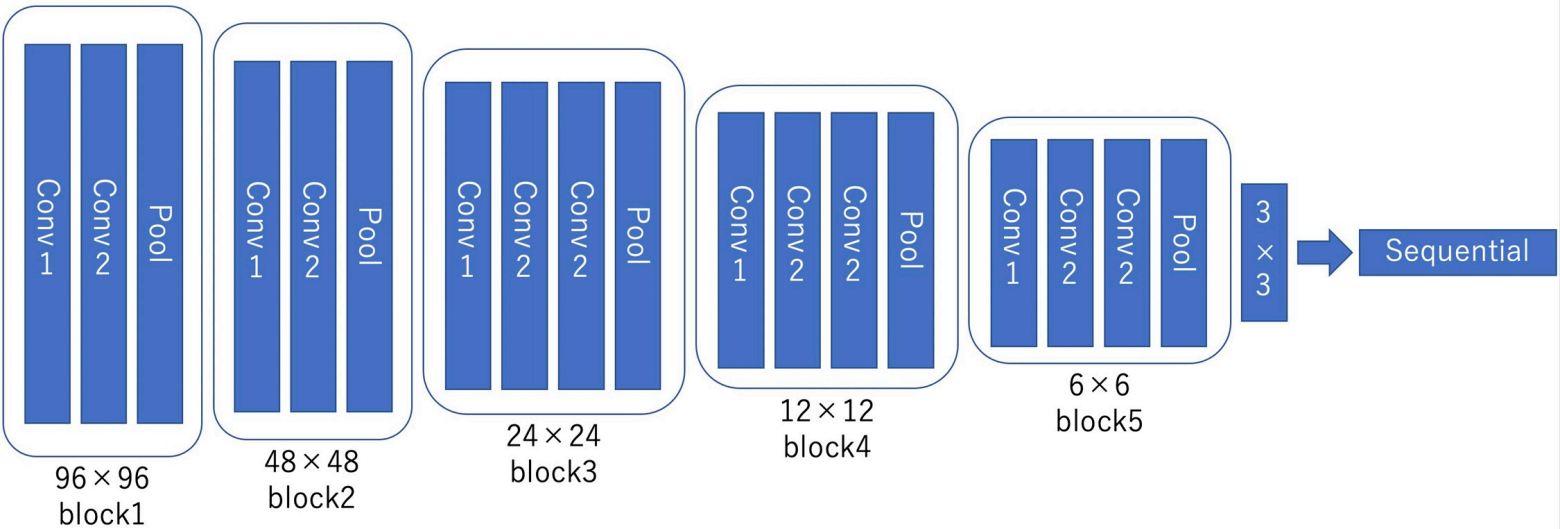
Scheme of the convolutional neural network.

The authors selected the VGG16 model because it has been trained on big data and its weights from the pre-training can be used by end-users from the internet to analyze data. The output data were compared with the initial teacher data (two categories–MTLE with the hippocampus as the epileptogenic area and controls). The deep learning model, which was a fine-tuned VGG16 model, comprised several layers (six convolutional layers, three maximum pooling layers, and three fully connected layers). Details of the neural network used in the model are shown in Fig 2. The number of epochs used for each study was 20. We used SGD as an optimizer for the deep learning model.

### 2.4. Visualization of deep learning

Explanation of the output of a deep network remains a challenge. The classification of images with AI is presented in the black box. Convolutional neural networks include multiple layers. An image classifier identifies pixels that have significant influence on the decision making. Therefore, we attempted to visualize the image processing using deep learning neural networks and determine the mechanisms used to classify or diagnose based on an image in the validation dataset. Here, we focused on the last layer to visualize the area of interest used by AI. One such visualization method for deep learning is the VGG16-based gradient-weighted class activation mapping (Grad-CAM) [10]. We used the Grad-CAM method to visualize the areas of interest to distinguish between MTLE and control using the AI program.

### 2.5. Diagnosis by six board-certified neurosurgeons

**Fig 3:**
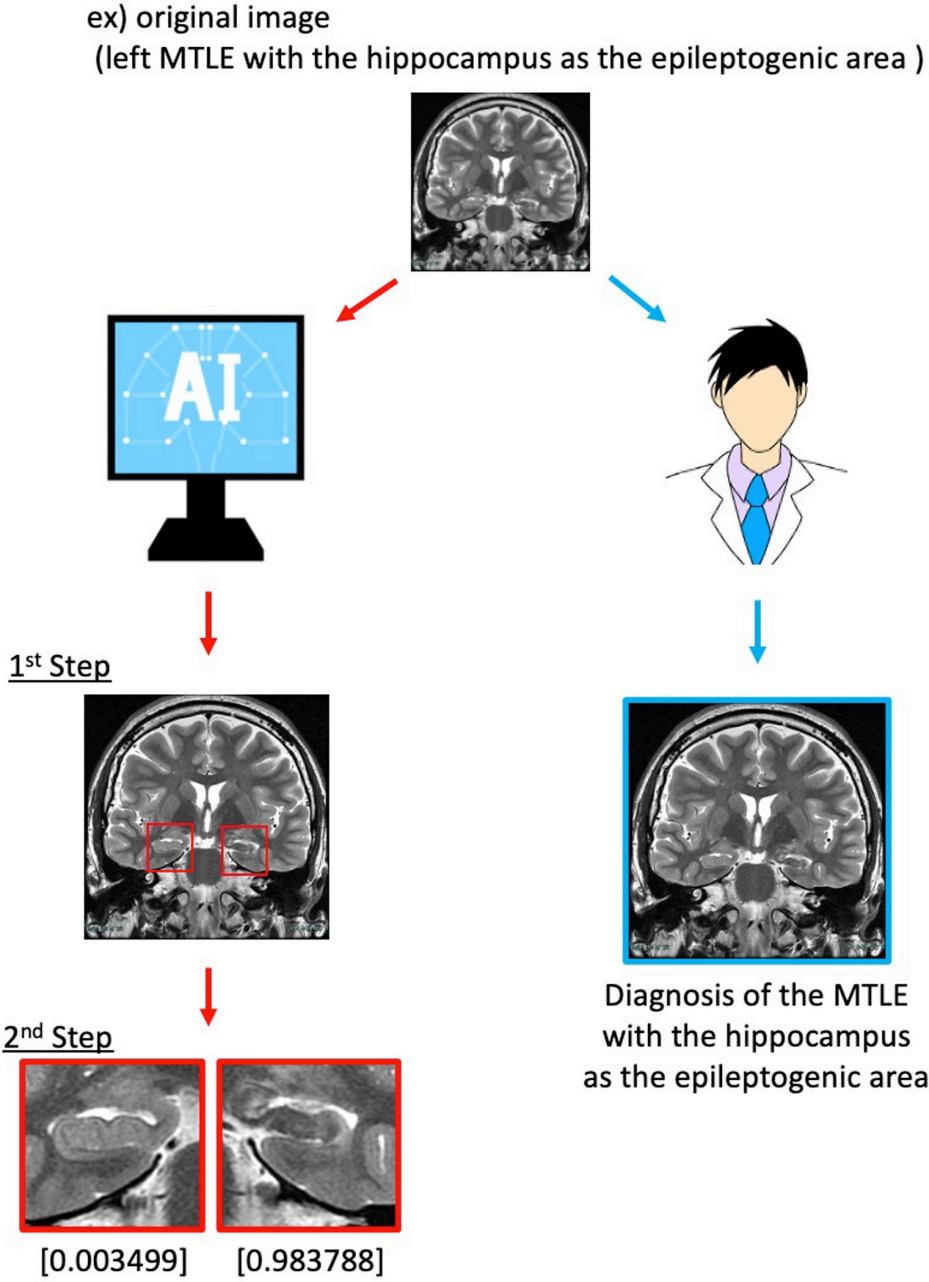
Scheme of hippocampal detection and prediction of MTLE with the hippocampus as the epileptogenic area. In the AI program, the first step is to automatically detect the hippocampus on conventional T2WI and FLAIR coronal sections. The second step is to diagnose MTLE with the hippocampus as the epileptogenic area and predict the probability of the disease. Six board-certified neurosurgeons diagnosed MTLE with the hippocampus as the epileptogenic area using an original image of one slice.

The analysis and diagnosis of MTLE based on T2 and FLAIR were performed by six independent investigators who were board-certified neurosurgeons (Fig 3). HS was diagnosed based on the presence of following characteristics: hippocampal atrophy, hippocampal hyperintensity on T2WI, and other hippocampal signal alterations such as loss of internal architecture of the hippocampus [11,12]. Three epilepsy specialists independently confirmed the diagnosis of MTLE with the hippocampus as the epileptogenic area based on MRI and EEG data.

#### 2.5.1. Selected images

We selected 20 MRI images for T2WI and FLAIR (10 images of patients diagnosed with MTLE with the hippocampus as the epileptogenic area and 10 patients diagnosed with other epilepsies). These 20 images represented the data of the group (Table 3).

**Table 3:** Summary of the findings of the T2-weighted and fluid-attenuated inversion recovery images of the 20 patients.

T2 number	FLAIR number	sex	age	laterality	diagnosis	EEG findings (spikes regional)	symptoms, seizure type
1	1	F	37	left	MTLE with the hippocampus as the epileptogenic area	left temporal	left conjugate deviation, oral automatism
2	2	F	39	left	MTLE with the hippocampus as the epileptogenic area	left temporal	abdominal aura, aphasic
3	3	M	9	left	MTLE with the hippocampus as the epileptogenic area	left temporal & spike and wave generalized	dialeptic seizure, oral automatism, automotor seizure
4	4	M	27	left	MTLE with the hippocampus as the epileptogenic area	left front-temporal	olfactory aura, dialeptic seizure, GTCS
5		F	22	left	MTLE with the hippocampus as the epileptogenic area	left temporal	dialeptic seizure, GTCS
	5	M	22	right	MTLE with the hippocampus as the epileptogenic area	right temporal	dialeptic seizure, automotor seizure
6		F	20	left	MTLE with the hippocampus as the epileptogenic area	left temporal	abdominal aura, dialeptic seizure, focal impaired awareness seizure
	6	M	17	right	MTLE with the hippocampus as the epileptogenic area	right front-temporal	autonomic aura, left versive, dialeptic seizure, left arm tonic seizure
7		M	22	right	MTLE with the hippocampus as the epileptogenic area	right temporal	dialeptic seizure, automotor seizure
8	7	M	42	right	MTLE with the hippocampus as the epileptogenic area	right temporal	abdominal aura, dialeptic seizure, automotor seizure
	8	F	45	right	MTLE with the hippocampus as the epileptogenic area	right and left temporal	abdominal aura, automotor seizure
9	9	M	30	right	MTLE with the hippocampus as the epileptogenic area	right front-temporal	dialeptic seizure
10		M	38	right	MTLE with the hippocampus as the epileptogenic area	right front-temporal	dialeptic seizure, GTCS
	10	F	22	right	MTLE with the hippocampus as the epileptogenic area	right temporal	automotor seizure
11		F	32	right	EBV encephalitis	right front-temporal	GTCS
12		M	33		Lennox-Gastaut syndrome	left temporal & sharp-and-slow-wave generalized	Tonic seizure, infantile spasm
13		F	29		Juvenile myoclonic epilepsy	generalized	dialeptic seizure, GTCS
14		M	48	right	right TLE	right temporal & paroxysmal fast lateralized right	abdominal aura, dialeptic seizure
15		F	30		PNES	no epileptic discharge	GTCS
16		F	35		right TLE	right temporal	GTCS
17		M	61	right	meningioma	no epileptic discharge	Tonic seizure, dialectic seizure
18		F	22		Juvenile myoclonic epilepsy	spike-and-wave complex &polyspike generalized	dialeptic seizure
19		F	57	left	left TLE, left amygdala hypertrophy	no epileptic discharge	dialeptic seizure, oral automatism
20		F	7	right	right OLE	right occipital	blindness
	11	M	20		meningioma, epilepsy	no epileptic discharge	GTCS
	12	M	14	right	right TLE, cavernoma	spikes regional right front-temporal	dialeptic seizure, automotor seizure
	13	M	36	left	left TLE	sharp wave regional left temporal	unclassified aura, automotor seizure, GTCS
	14	M	21	left	epilepsy	sharp wave lateralized left	bilateral asymmetric tonic, GTCS
	15	F	16		PNES	no epileptic discharge	dialeptic seizure
	16	M	18	right	right TLE, right amygdala hypertrophy	spikes regional right temporal	dialeptic seizure, GTCS
	17	M	67	left	left TLE, left amygdala hypertrophy	sharp wave regional left temporal	dialeptic seizure, automotor seizure
	18	M	25		PNES	no epileptic discharge	twitching, stop working
	19	F	5	right	right FLE	spikes regional right frontal and occipital	bilateral arm tonic seizure, dialeptic seizure
	20	M	19	right	left TLE	repetitive spike regional right fronto-temporal	autonomic aura, dialeptic, GTCS

20 patientws including 10 MTLE with the hippocampus as the epileptogenic area (case 1–10) and 10 non-MTLE with the hippocampus as the epileptogenic area (case 11–20).

MTLE, mesial temporal lobe epilepsy; GTCS, generalized tonic-clonic convulsion; PNES, psychogenic non-epileptic seizures.

#### 2.5.2. Patients diagnosed with MTLE

Retrospectively, 46 patients were diagnosed with MTLE at our institution. However, we excluded cases with no imaging, cases in which surgery was performed, and cases for which no ECoG recording with subdural electrodes existed. Finally, T2WI was validated in 28 cases and FLAIR in 34 cases.

### 2.6. Statistical analysis

Data are presented as mean ± standard deviation or standard error. Statistical analysis was performed using IBM SPSS 22.0 (IBM Corp. Armonk, NY, USA). The Shapiro–Wilk test was used to confirm whether the data followed a normal distribution.When the data distribution was not normal, statistical differences were assessed using the Mann-Whitney U test. We also performed a cross tabulation. A p value of <0.01 was considered statistically significant.

## 3. Results

### 3.1. Stratification of the patients

This study enrolled 12 patients who were diagnosed with MTLE with the hippocampus as the epileptogenic area based on MRI and EEGs by three epilepsy specialists. To assess the diagnostic accuracy, the patients were divided into two groups: those with MTLE with the hippocampus as the epileptogenic area and control subjects. Imaging data of both the groups were further divided into the learning and validation datasets. The validation dataset comprised approximately 30% the data of both the groups (patients with MTLE with the hippocampus as the epileptogenic area and control subjects). The validation dataset included data of randomly selected patients. Patients’ data in the validation and learning datasets were statistically matched for age and sex.

Bilateral hippocampi were automatically and successfully detected by the AI program, with an accuracy of 96%–99% using T2WI and of 89% using FLAIR images.

Haar-like feature was selected because it is more accurate than LBP when the hyperparameters are changed to optimize the detection rate and reduce false positives. FLAIR had a 89% detection rate and 0.25 false positives per picture. While checking the detection rate and false positive rate, we examined the effect of changing mainly the following parameters as optimization factors: scaleFactor, minNeighbors, minSize, and maxSize. For the T2WI classifier, scaleFactor was set to 1.06, minNeighbors to 3, minSize to (60,60), and maxSize to (130,130). Similarly, for FLAIR, we set the scaleFactor to 1.1202, minNeighbors to 3, minSize to (60,60), and maxSize to (130,130).

**Fig 4:**
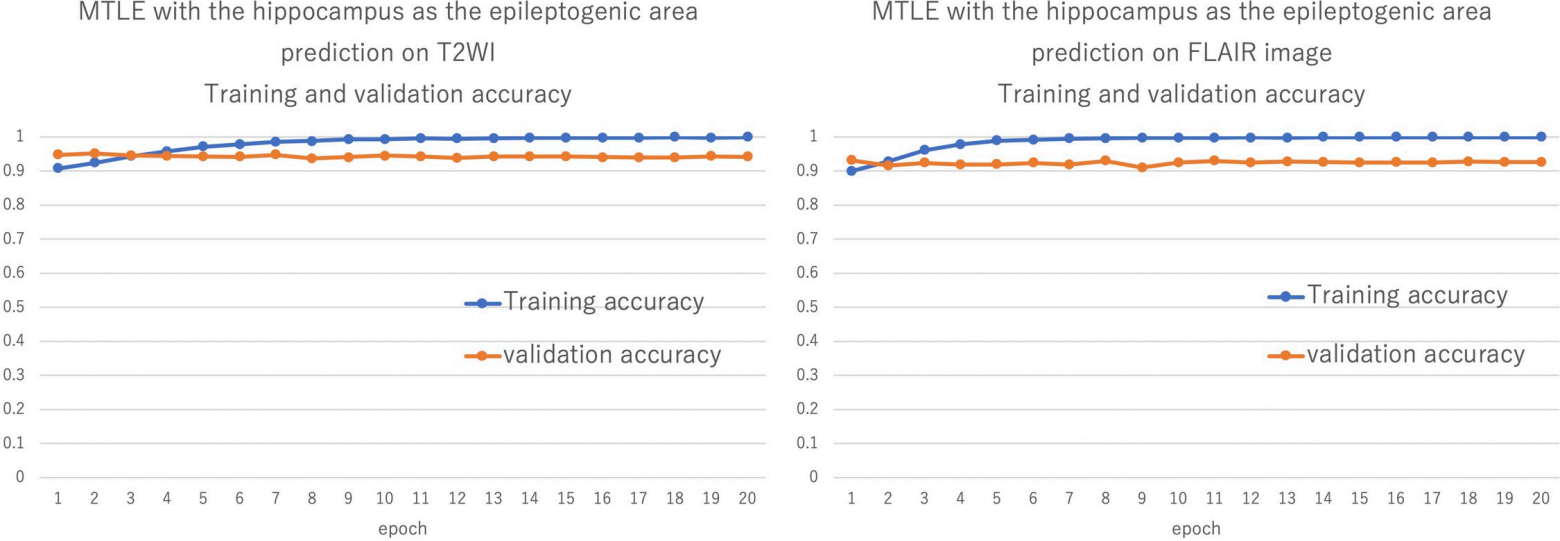
Validation of overall diagnostic accuracy. The diagnostic accuracy of the deep learning model for MTLE with the hippocampus as the epileptogenic area based on T2WI was 94.13% (left) and on FLAIR was 94.91% (right).

We compared the diagnoses of MTLE with the hippocampus as the epileptogenic area between six board-certified neurosurgeons and AI for each MRI sequence (T2WI and FLAIR). The diagnostic accuracy of AI using T2WI data was 94% and using FLAIR data was 95% (Fig 4). When the diagnostic accuracy was measured using only the original images as the test data, the diagnostic accuracy using T2WI data was 98%, and that using FLAIR data was 95%. However, this result is considered to be insufficiently evaluated because of the small number of original images.

We performed a cross-validation analysis, and the system showed 90–93% accuracy for the T2WI data and 89–95% accuracy for the FLAIR data. For MTLE with the hippocampus as the epileptogenic area, 3 out of 9 T2 cases and 10 FLAIR cases were selected as validation data. Verification was performed five times by replacing the data. Allocation was done using a random number table. The relatively high accuracy from the beginning of the epoch may be due firstly to the fact that VGG16 was a very good model for MTLE differentiation, and secondly to the fact that the image size was small (96 × 96 pixels) and the accuracy was high. However, considering that diagnostic accuracy increased with each successive epoch, we believe that there was a learning effect.

### 3.2. Selected images

The disease (MTLE with the hippocampus as the epileptogenic area) probability in the extracted hippocampus was found to be 92.5% based on T2WI and 82.5% based on FLAIR using the AI program. On the other hand, the disease (MTLE with the hippocampus as the epileptogenic area) probability was 74.1% based on T2WI and 74.2% based on FLAIR according to the six board-certified neurosurgeons. Those judged to have a 50% or greater likelihood of hippocampal sclerosis, according to the AI diagnosis, were treated as correct answers. Those that were not hippocampal sclerosis were treated as correct if the likelihood of hippocampal sclerosis was judged to be less than 50%. Interestingly, diagnosis with AI was good in all, except one, cases; one of the 20 images showed false positives on T2WI and seven of the 20 images on FLAIR. Diagnoses by the board-certified neurosurgeons based on T2 and FLAIR were not different.

The images for which MTLE with the hippocampus as the epileptogenic area was misdiagnosed as normal, or *vice-versa*, always showed a tendency towards hyperintensity on T2WI. Diagnoses by AI were significantly more accurate than those by board-certified neurosurgeons based on T2WI images (p = 0.0001, effect size 0.6152); however, the difference in diagnostic accuracies of AI and neurosurgeons based on FLAIR was not statistically significant (p = 0.0520, effect size 0.3072) (Table 4).

**Table 4:** Diagnostic accuracy of artificial intelligence & neurosurgeons.

	FLAIR			T2WI		
case	AI	Neurosurgeons	(p = 0.0520)	AI	Neurosurgeons	(p = 0.0001)
1	0.95	0.67		0.99	0.50	
2	0.25	0.83		1.00	0.17	
3	0.90	1.00		0.91	0.83	
4	0.01	0.33		0.98	0.83	
5	0.02	0.33		0.77	0.50	
6	0.00	0.83		1.00	0.67	
7	0.06	0.50		0.99	0.17	
8	0.25	0.50		0.01	0.50	
9	0.07	0.17		0.95	0.50	
10	0.23	0.67		0.04	0.33	
11	0.00	0.50		0.00	0.50	
12	0.00	0.83		0.00	0.33	
13	0.00	0.33		0.00	0.83	
14	0.00	0.83		0.01	0.83	
15	0.00	0.83		0.00	0.33	
16	0.02	0.67		0.00	0.83	
17	0.01	0.00		0.00	0.50	
18	0.00	0.33		0.48	0.67	
19	0.00	0.83		0.01	0.33	
20	0.01	0.67		0.05	0.50	

20 patientws including 10 MTLE with the hippocampus as the epileptogenic area (case 1–10) and 10 non-MTLE with the hippocampus as the epileptogenic area patients (case 11–20).

However, the sensitivity, specificity, and F-value of AI for T2WI were 0.8000, 0.9667, and 0.8421, respectively, and those of physicians for T2WI were 0.5000, 0.5667, and 0.5172, respectively. Similarly, for FLAIR, the sensitivity, specificity, and F-value for the diagnosis by AI were 0.3077, 1.0000, and 0.4706, respectively, whereas those by physicians were 0.5833, 0.5833, and 0.5833, respectively. Although the sensitivity, specificity, and F-value of AI for T2WI were high, the sensitivity for FLAIR was low.

### 3.3. Patients diagnosed with MTLE

The Shapiro–Wilk test showed that the data did not follow a normal distribution (p<0.001); therefore, Mann–Whitney U test was performed. The diagnostic accuracy for MTLE by AI was higher than that by physicians for both T2WI and FLAIR (T2, p = 0.0034; effect size 0.3917, FLAIR, p = 0.0006; effect size, 0.4147). AI diagnosis was superior in the cross tabulation (p<0.001) as well.

However, the sensitivity, specificity, and F-value of AI for T2WI were 0.5556, 0.9362, and 0.5582, respectively, and those of physicians for T2WI were 0.5000, 0.6754, and 0.4576, respectively. Similarly, in FLAIR, the sensitivity, specificity, and F-value for the diagnosis by AI were 0.4, 1.0000, and 0.5714, respectively, whereas those by physicians were 0.6833, 0.6181, and 0.5256, respectively. Although the F-value for AI was high, the sensitivity was low.

### 3.4. Diagnostic site of MTLE with the hippocampus as the epileptogenic area

**Fig 5:**
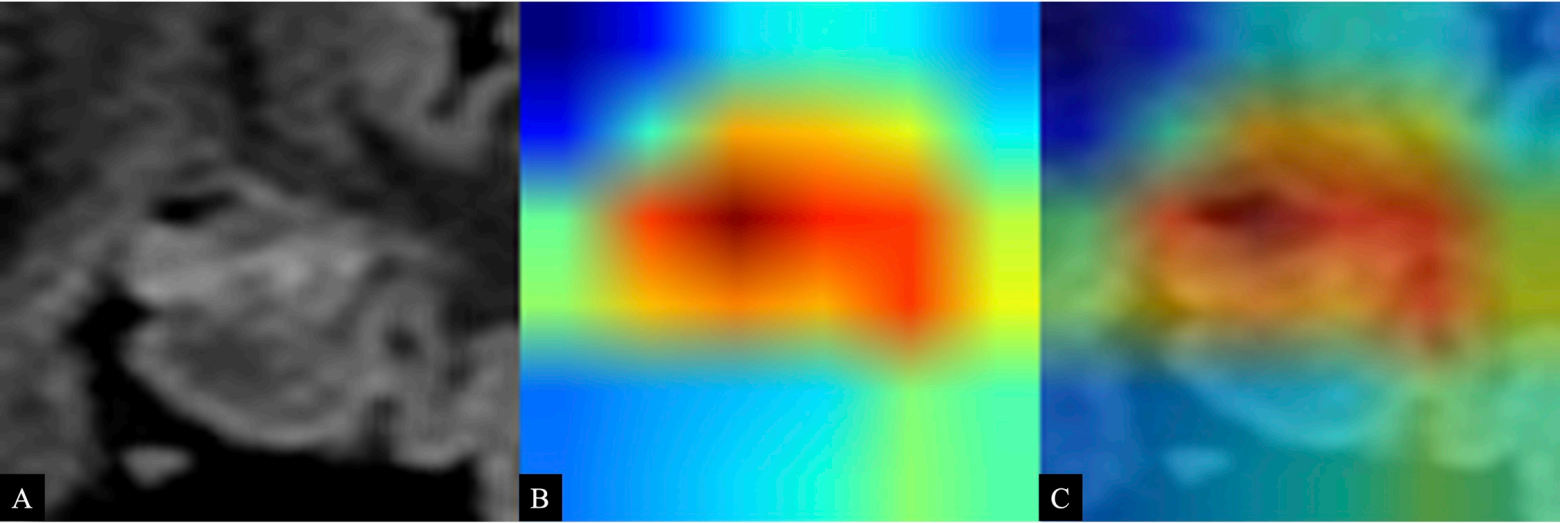
Visualization of the area of interest by deep learning. FLAIR image of a patient with MTLE with the left hippocampus as the epileptogenic area showing abnormal hyperintensity and atrophy (A). Visualization with color mapping using Grad-CAM technique (B) and fusion (C); areas of interest and “hot areas” crowded together surrounding the hippocampus and parahippocampal area.

AI focused on the color gradient of the area of interest for diagnosing the epileptogenic area in the hippocampus (Fig 5). The focus was expected to be on the outer area as well as on the surface of the hippocampus based on the usual pathology.

## 4. Discussion

The occurrence of MTLE with HS is rare. It originates from the limbic system in the mesial temporal lobe, particularly the hippocampus, amygdala, and parahippocampal gyrus, as well as their connections. HS is characterized pathologically by prominent neuronal loss and gliosis in the hippocampus and amygdala [13]. It is crucial to determine whether the seizure originates from the medial, lateral, or multifocal epileptogenic points because the diagnosis is difficult and therapeutic methods for each condition differ. The curative effect of the surgical treatment is well-established because the attack resolution rate after medical therapy is only 8% per year, whereas that after surgical treatment is 58% in the case of intractable TLE [14]. In addition, it is necessary to localize the epileptic focus preoperatively in several cases to prevent unnecessary brain resection. After the detailed localization of an epileptogenic focus through cortical mapping, certain invasive examinations such as cortical mapping with subdural electrodes may be required before moving on to resection as a second-stage surgery. However, it would be remarkable if a noninvasive examination could guide the diagnosis and localization of an epileptogenic focus.

If HS can be diagnosed preoperatively using noninvasive modalities such as MRI, a temporal lobectomy can be performed in a single stage [15–17]. Jack et al. (Mayo Clinic, 1990) noninvasively showed hippocampal atrophy and sclerotic change in an MRI coronal section; thereafter, assessing etiology, clinical course characteristics, EEG data, convalescence, surgical outcomes, and prognosis has become easy [18]. In 1993, Wieser indicated that MTLE is equivalent to an epileptic syndrome or as a disease unit [19]. Although MTLE is intractable, even with treatment using multiple anticonvulsant agents, it can be well-distinguished from their epileptic syndromes such as lateral TLE since they show surgical outcomes. Therefore, differential diagnosis is very important.

The epidemiological cause of MTLE is HS, accounting for approximately two-thirds of all cases. Other causes include neoplastic lesions (such as ganglioglioma, glioma, and dysembryoplastic neuroepithelial tumor) and vascular lesions (such as arteriovenous malformations, cavernous angiomas or venous angiomas, and cortical dysplasia) [20]. Furthermore, MTLE associated with HS predominantly occurs among individuals aged is 4–16 years. MTLE due to HS generally occurs earlier than MTLE due to other causes [21].

MTLE is often associated with anamneses such as febrile convulsion (particularly complicated), brain hypoxia, an infectious disease, and/or head injury [21].

Hippocampal dysfunction initiates epileptogenic seizures and impairs cognition in patients with MTLE [22].

Recently, the use of AI in the medical care field has increased at a tremendous pace. For example, a patient was diagnosed with leukemia using the AI program within only 10 minutes after learning from more than 20 million cancer research articles, which proved lifesaving (Watson, IBM co.).

Several studies have presented a machine learning-based method for identifying the epileptic seizure onset zone [23–29]. The reason for the improvement is technical skillfulness and precise evaluation of the epileptogenic zone in the brain alongside functional mapping.

Recently, many scholars have been engaged in hippocampus segmentation algorithms. A number of methods have been proposed, including cluster methods [30], using map and feature embedding models or iterative local linear mapping models [31], atlas-based and label fusion methods [32], statistical machine learning techniques combined with KNN and SVM models [33], probabilistic modeling frame methods [34], nonlinear image registration algorithms [35], energy minimization models [36], using local Gaussian distribution fitting energy with level set function and local mean and variance as variables [37,38], and bias-corrected distance regularized level set method with MR image contrast enhancement [39].

Based on the aforementioned example, AI is suitable for processing a large amount of medical data (such as diagnostic imaging data), comparing case data with previous research data, and organizing data. Moreover, AI is a developing technology in the medical field. Previous data on rare diseases such as neurosurgical diseases are limited; hence, the accuracy may not be reliable because AI diagnosis requires a large amount of data. Here, we demonstrated an example of a diagnostic imaging technique for epilepsy.

As shown in Fig 5, AI focuses on the CA1 and CA2 regions of the hippocampus. In hippocampal sclerosis, pathological findings include not only neuronal atrophy but also neuronal loss and gliosis. Although not beyond the realm of conjecture, it is possible that AI can identify tiny differences in MRI signal values that cannot be corroborated by the physician’s naked eye. We believe that the results of this study are helpful to specialists as well as non-specialists in community medicine for aiding in the diagnosis of epilepsy.

## 5. Limitations

Recently, deep learning technology has advanced rapidly; however, its ability to simultaneously diagnose several cases is suboptimal. In addition, the possibility of misdiagnosis because of bugs or malfunctioning of the software is inevitable. AI in medical care remains on the periphery of diagnostic science. Further research is required to learn and push the boundaries of the field.

## 6. Conclusion

We present a highly accurate deep learning-based AI program that can diagnose MTLE with the hippocampus as the epileptogenic area better than some board-certified neurosurgeons.

Almost all AI programs that are big hit worldwide are not all-purpose and perform limited functions such as extract and reproduce human specific data on the computer. However, AI is a powerful tool and can sometimes overcome the biases shown by humans. In addition, AI has the advantage of generating stable results. In the medical field, doctors have harsh working environments, which include overwork due to lack of personnel, frequent calls at night, and insufficient holidays; this may account for false judgments sometimes. On the contrary, AI can maintain a certain level of output accuracy and be a reliable assistant to doctors.

## References

[1] Penfield W. Temporal lobe epilepsy. Br J Surg. 1954;41(168):337–43. doi: 10.1002/bjs.18004116802 . 13126468

[2] Sander JW. Some aspects of prognosis in the epilepsies: a review. Epilepsia. 1993;34(6):1007–16. doi: 10.1111/j.1528-1157.1993.tb02126.x . 8243349

[3] Blakemore CB, Falconer MA. Long-term effects of anterior temporal lobectomy on certain cognitive functions. J Neurol Neurosurg Psychiatry. 1967;30(4):364–7. doi: 10.1136/jnnp.30.4.364 ; PubMed Central PMCID: PMC496201. 6055346 PMC496201

[4] Scoville WB, Milner B. Loss of recent memory after bilateral hippocampal lesions. 1957. J Neuropsychiatry Clin Neurosci. 2000;12(1):103–13. doi: 10.1176/jnp.12.1.103 . 10678523

[5] Zarow C, Sitzer TE, Chui HC. Understanding hippocampal sclerosis in the elderly: epidemiology, characterization, and diagnostic issues. Curr Neurol Neurosci Rep. 2008;8(5):363–70. doi: 10.1007/s11910-008-0057-3 . 18713571

[6] Bower SP, Vogrin SJ, Morris K, Cox I, Murphy M, Kilpatrick CJ, et al. Amygdala volumetry in "imaging-negative" temporal lobe epilepsy. J Neurol Neurosurg Psychiatry. 2003;74(9):1245–9. doi: 10.1136/jnnp.74.9.1245 ; PubMed Central PMCID: PMC1738652. 12933928 PMC1738652

[7] Barba C, Barbati G, Minotti L, Hoffmann D, Kahane P. Ictal clinical and scalp-EEG findings differentiating temporal lobe epilepsies from temporal ’plus’ epilepsies. Brain. 2007;130(Pt 7):1957–67. Epub 2007/05/31. doi: 10.1093/brain/awm108 . 17535836

[8] Blumcke I, Thom M, Aronica E, Armstrong DD, Bartolomei F, Bernasconi A, et al. International consensus classification of hippocampal sclerosis in temporal lobe epilepsy: a Task Force report from the ILAE Commission on Diagnostic Methods. Epilepsia. 2013;54(7):1315–29. Epub 2013/05/23. doi: 10.1111/epi.12220 . 23692496

[9] Jack CR Jr., Rydberg CH, Krecke KN, Trenerry MR, Parisi JE, Rydberg JN, et al. Mesial temporal sclerosis: diagnosis with fluid-attenuated inversion-recovery versus spin-echo MR imaging. Radiology. 1996;199(2):367–73. Epub 1996/05/01. doi: 10.1148/radiology.199.2.8668780 . 8668780

[10] Simonyan K, Zisserman A. Very Deep Convolutional Networks for Large-Scale Image Recognition. 3rd ed. Conf Track Proc 20152015.

[11] Bronen RA, Fulbright RK, Kim JH, Spencer SS, Spencer DD, al-Rodhan NR. Regional distribution of MR findings in hippocampal sclerosis. AJNR Am J Neuroradiol. 1995;16(6):1193–200. ; PubMed Central PMCID: PMC8337833. 7677010 PMC8337833

[12] Oppenheim C, Dormont D, Biondi A, Lehéricy S, Hasboun D, Clémenceau S, et al. Loss of digitations of the hippocampal head on high-resolution fast spin-echo MR: a sign of mesial temporal sclerosis. AJNR Am J Neuroradiol. 1998;19(3):457–63. ; PubMed Central PMCID: PMC8338269. 9541299 PMC8338269

[13] Williamson PD, French JA, Thadani VM, Kim JH, Novelly RA, Spencer SS, et al. Characteristics of medial temporal lobe epilepsy: II. Interictal and ictal scalp electroencephalography, neuropsychological testing, neuroimaging, surgical results, and pathology. Ann Neurol. 1993;34(6):781–7. doi: 10.1002/ana.410340605 . 8250526

[14] Wiebe S, Blume WT, Girvin JP, Eliasziw M. A randomized, controlled trial of surgery for temporal-lobe epilepsy. N Engl J Med. 2001;345(5):311–8. doi: 10.1056/NEJM200108023450501 . 11484687

[15] Kilpatrick C, Cook M, Kaye A, Murphy M, Matkovic Z. Non-invasive investigations successfully select patients for temporal lobe surgery. J Neurol Neurosurg Psychiatry. 1997;63(3):327–33. doi: 10.1136/jnnp.63.3.327 ; PubMed Central PMCID: PMC2169725. 9328249 PMC2169725

[16] Sperling MR, O’Connor MJ, Saykin AJ, Phillips CA, Morrell MJ, Bridgman PA, et al. A noninvasive protocol for anterior temporal lobectomy. Neurology. 1992;42(2):416–22. doi: 10.1212/wnl.42.2.416 . 1736176

[17] Takayama M, Miyamoto S, Ikeda A, Mikuni N, Takahashi JB, Usui K, et al. Intracarotid propofol test for speech and memory dominance in man. Neurology. 2004;63(3):510–5. doi: 10.1212/01.wnl.0000133199.65776.18 . 15304583

[18] Jack CR Jr., Sharbrough FW, Twomey CK, Cascino GD, Hirschorn KA, Marsh WR, et al. Temporal lobe seizures: lateralization with MR volume measurements of the hippocampal formation. Radiology. 1990;175(2):423–9. doi: 10.1148/radiology.175.2.2183282 . 2183282

[19] Wieser H, Engel J, Williamson PD. Surgically remediable temporal lobe syndromes. 2nd ed. New York: Raven Press 1993.

[20] Tatum WOt. Mesial temporal lobe epilepsy. J Clin Neurophysiol. 2012;29(5):356–65. doi: 10.1097/WNP.0b013e31826b3ab7 . 23027091

[21] Wieser HG. ILAE Commission Report. Mesial temporal lobe epilepsy with hippocampal sclerosis. Epilepsia. 2004;45(6):695–714. doi: 10.1111/j.0013-9580.2004.09004.x . 15144438

[22] Engel J Jr. Mesial temporal lobe epilepsy: what have we learned? Neuroscientist. 2001;7(4):340–52. doi: 10.1177/107385840100700410 . 11488399

[23] Akter MS, Islam MR, Iimura Y, Sugano H, Fukumori K, Wang D, et al. Multiband entropy-based feature-extraction method for automatic identification of epileptic focus based on high-frequency components in interictal iEEG. Sci Rep. 2020;10(1):7044. Epub 2020/04/29. doi: 10.1038/s41598-020-62967-z ; PubMed Central PMCID: PMC7184764. 32341371 PMC7184764

[24] Baud MO, Kleen JK, Anumanchipalli GK, Hamilton LS, Tan YL, Knowlton R, et al. Unsupervised Learning of Spatiotemporal Interictal Discharges in Focal Epilepsy. Neurosurgery. 2018;83(4):683–91. Epub 2017/10/19. doi: 10.1093/neuros/nyx480 ; PubMed Central PMCID: PMC6454796. 29040672 PMC6454796

[25] Elahian B, Yeasin M, Mudigoudar B, Wheless JW, Babajani-Feremi A. Identifying seizure onset zone from electrocorticographic recordings: A machine learning approach based on phase locking value. Seizure. 2017;51:35–42. Epub 2017/08/05. doi: 10.1016/j.seizure.2017.07.010 . 28772200

[26] Grinenko O, Li J, Mosher JC, Wang IZ, Bulacio JC, Gonzalez-Martinez J, et al. A fingerprint of the epileptogenic zone in human epilepsies. Brain. 2018;141(1):117–31. Epub 2017/12/19. doi: 10.1093/brain/awx306 ; PubMed Central PMCID: PMC5837527. 29253102 PMC5837527

[27] Murin Y, Kim J, Parvizi J, Goldsmith A. SozRank: A new approach for localizing the epileptic seizure onset zone. PLoS Comput Biol. 2018;14(1):e1005953. Epub 2018/01/31. doi: 10.1371/journal.pcbi.1005953 ; PubMed Central PMCID: PMC5806930. 29381703 PMC5806930

[28] Nissen IA, Stam CJ, van Straaten ECW, Wottschel V, Reijneveld JC, Baayen JC, et al. Localization of the Epileptogenic Zone Using Interictal MEG and Machine Learning in a Large Cohort of Drug-Resistant Epilepsy Patients. Front Neurol. 2018;9:647. Epub 2018/08/23. doi: 10.3389/fneur.2018.00647 ; PubMed Central PMCID: PMC6090046. 30131762 PMC6090046

[29] Varatharajah Y, Berry B, Cimbalnik J, Kremen V, Van Gompel J, Stead M, et al. Integrating artificial intelligence with real-time intracranial EEG monitoring to automate interictal identification of seizure onset zones in focal epilepsy. J Neural Eng. 2018;15(4):046035. Epub 2018/06/02. doi: 10.1088/1741-2552/aac960 ; PubMed Central PMCID: PMC6108188. 29855436 PMC6108188

[30] Pang S, Jiang J, Lu Z, Li X, Yang W, Huang M, et al. Hippocampus Segmentation Based on Local Linear Mapping. Sci Rep. 2017;7:45501. Epub 20170403. doi: 10.1038/srep45501 ; PubMed Central PMCID: PMC5377362. 28368016 PMC5377362

[31] Pang S, Lu Z, Jiang J, Zhao L, Lin L, Li X, et al. Hippocampus Segmentation Based on Iterative Local Linear Mapping With Representative and Local Structure-Preserved Feature Embedding. IEEE Trans Med Imaging. 2019;38(10):2271–80. Epub 20190321. doi: 10.1109/TMI.2019.2906727 . 30908202

[32] Carmichael OT, Aizenstein HA, Davis SW, Becker JT, Thompson PM, Meltzer CC, et al. Atlas-based hippocampus segmentation in Alzheimer’s disease and mild cognitive impairment. Neuroimage. 2005;27(4):979–90. doi: 10.1016/j.neuroimage.2005.05.005 ; PubMed Central PMCID: PMC2862692. 15990339 PMC2862692

[33] Hao Y, Wang T, Zhang X, Duan Y, Yu C, Jiang T, et al. Local label learning (LLL) for subcortical structure segmentation: application to hippocampus segmentation. Hum Brain Mapp. 2014;35(6):2674–97. Epub 20131023. doi: 10.1002/hbm.22359 ; PubMed Central PMCID: PMC6869539. 24151008 PMC6869539

[34] Platero C, Tobar MC, Sanguino J, Velasco O, editors. A New Label Fusion Method Using Graph Cuts: Application to Hippocampus Segmentation 2014; Cham: Springer International Publishing.

[35] Zhu H, Tang Z, Cheng H, Wu Y, Fan Y. Multi-atlas label fusion with random local binary pattern features: Application to hippocampus segmentation. Sci Rep. 2019;9(1):16839. Epub 20191114. doi: 10.1038/s41598-019-53387-9 ; PubMed Central PMCID: PMC6856174. 31727982 PMC6856174

[36] van der Lijn F, den Heijer T, Breteler MM, Niessen WJ. Hippocampus segmentation in MR images using atlas registration, voxel classification, and graph cuts. Neuroimage. 2008;43(4):708–20. Epub 20080812. doi: 10.1016/j.neuroimage.2008.07.058 . 18761411

[37] Jiang X, Zhou Z, Ding X, Deng X, Zou L, Li B. Level Set Based Hippocampus Segmentation in MR Images with Improved Initialization Using Region Growing. Comput Math Methods Med. 2017;2017:5256346. Epub 20170115. doi: 10.1155/2017/5256346 ; PubMed Central PMCID: PMC5274694. 28191031 PMC5274694

[38] Wang L, Chen Y, Pan X, Hong X, Xia D. Level set segmentation of brain magnetic resonance images based on local Gaussian distribution fitting energy. J Neurosci Methods. 2010;188(2):316–25. Epub 20100315. doi: 10.1016/j.jneumeth.2010.03.004 . 20230858

[39] Selma T, Madusanka N, Kim T-H, Kim Y-H, Mun C-W, Choi H-K. Contrast-enhanced Bias-corrected Distance-regularized Level Set Method Applied to Hippocampus Segmentation. Journal of Korea Multimedia Society. 2016;19(8):1236–47. doi: 10.9717/kmms.2016.19.8.1236

