# Supplementary material for: Deep learning for the diagnosis of mesial temporal lobe epilepsy
Source: PLoS One. 2023 Feb 23;18(2):e0282082. doi: 10.1371/journal.pone.0282082 (PMC9949622; doi:10.1371/journal.pone.0282082)
Supplement: Attachment — Submitted filename: renamed_0ec42.docx [file pone.0282082.s002.docx]

October 26, 2022

Dear Dr. Emily Chenette:

Thank you for your thoughtful and constructive feedback regarding our manuscript titled “Deep Learning for the Diagnosis of Mesial Temporal Lobe Epilepsy.”

We also appreciate the time and effort you and each of the reviewers have dedicated to providing suggestions, which have strengthened our paper. Thus, it is with great pleasure that we resubmit our article for further consideration. We have incorporated changes that reflect your suggestions. We hope that our edits and responses satisfactorily address all of the noted issues and concerns.

Reviewer #1:

• Please elaborate the statement that the “diagnostic accuracy is increased in a successive epoch”. As observed from the Figure 4, the validation accuracy is decreasing or remaining the same throughout the training. The training accuracy is expected to increase and saturate overtime. The validation accuracy typically represents the results on an unknown test set. Therefore, if the validation accuracy is high without any training, it means that the model without any training is can perform classification task. Ideally, the validation accuracy should increase with training and later saturate or decrease due to overfitting.

RESPONSE:

Thank you for your valuable comment. Although it is difficult to understand which epoch the horizontal axis is referring to in the graph as the number of iterations increases within 1 epoch, accuracy increases to about 0.8 within the 1^st^ epoch. In this learning, the accuracy almost reaches a plateau at 2 epochs, making this difficult to reflect it in the graph.

• Thank you for adding a Tables 2 & 3 regarding patient information. However, please combine these two tables since I found that most of the patients were overlapping between the two analyses.

The Table 1 is interesting. Please include details regarding the ‘validation’ set. Also, I find it consuming why data augmentation was performed on the test set. Data augmentation is performed on the training set to increase the number of observations and to make the system robust to noise. If it was applied on the test set, you are typically skewing the performance metrics. I understood you have presented both the results, but the results based on data augmentation is skewed.

RESPONSE:

Thank you for your valuable suggestion. We have compiled the data previously presented in Tables 2 and 3 in the new Table 3. The data in Table 1 has been revised, with the amplified validation data deleted. We have added the details regarding the validation data in the new Table 2. Cross-validation was performed using three of these cases as validation data.

• Please specify the details of cross-validation: How many folds? How was the hyper parameters optimized? etc.

RESPONSE:

For MTLE with the hippocampus as the epileptogenic area, three out of nine T2 cases and 10 FLAIR cases were selected as validation data. Verification was performed five times by replacing the data. We obtained results of 90–93% accuracy for T2WI and 89–95% accuracy for FLAIR.

• The reply to this question is incomplete. Please specify whether you have performed normality testing of the feature distribution to choose Mann-Whitney U test. Also, specify the type of d-values presented.

Now, the modification on p 12, line 288 have given more concerns regarding the statistical testing. Mann-Whitney U test is performed to assess if two distribution as significantly different. However, since the authors claim that the prediction values were converted to binary as correct or wrong, then this statistical testing cannot be applied.

RESPONSE:

Thank you for your valuable comment. The Shapiro–Wilk test showed that the data did not follow a normal distribution (p<0.000); therefore, Mann–Whitney U test was performed.

Thank you again for giving us the opportunity to strengthen our manuscript with your valuable comments and suggestions. We have worked hard to incorporate your feedback, and hope that our revised manuscript is suitable for publication.

Yours sincerely,

Kyoya Sakashita, M.D., Nobuhiro Mikuni, D.D., Ph.D.

Department of Neurosurgery

Sapporo Medical University School of Medicine

S1, W16 Chuo-ku Sapporo

Hokkaido 060-8543, Japan

Tel: +81 11-611-2111

Fax: +81 11-614-1662

Email: kyoya.s628@gmail.com
