# Supplementary material for: Deep learning for the diagnosis of mesial temporal lobe epilepsy
Source: PLoS One. 2023 Feb 23;18(2):e0282082. doi: 10.1371/journal.pone.0282082 (PMC9949622; doi:10.1371/journal.pone.0282082)
Supplement: Attachment — Submitted filename: renamed_2ccaa.docx [file pone.0282082.s003.docx]

December 23, 2022

Dear Dr. Emily Chenette:

Thank you for your thoughtful and constructive feedback regarding our manuscript titled “Deep Learning for the Diagnosis of Mesial Temporal Lobe Epilepsy.”

We also appreciate the time and effort you and each of the reviewers have dedicated to providing suggestions, which have strengthened our paper. Thus, it is with great pleasure that we resubmit our article for further consideration. We have incorporated changes that reflect your suggestions. We hope that our edits and responses satisfactorily address all of the noted issues and concerns.

Reviewer #1:

Question: This is exactly where I am confused. Within 2 epochs, the model saturated and the validation accuracy started decreasing. This just shows that the VGG16 model can detect MTLE even without re-training the model. This just shows that the entire training process is redundant in this study.

RESPONSE:

Thank you for pointing this out. We apologize for not being able to give a clear answer. To check whether the VGG16 model can detect MTLE, we performed 20 diagnoses that were also used in the paper with a 1-epoch learning model. The result was not limited to whether MTLE was true or false, and the probability of MTLE was between 0.10 and 0.41 for FLAIR and between 0.01 and 0.55 for T2, which were not diagnostically useful. Although it is not a direct answer, it shows that it is necessary to repeat the number of times of learning.

Question: Please elaborate on this procedure of replacing the data. Since this cross-validation results are the key results of the paper it needs to be well detailed.

RESPONSE:

A random number was used to assign which cases were to be trained and which were to be tested. (p.11, line 281)

| Validation case number  (case number of Table 4) | | Validation accuracy |
| --- | --- | --- |
| FLAIR | 1, 5, 6 | 0.8857 |
| FLAIR | 2, 3, 9 | 0.9250 |
| FLAIR | 1, 5, 10 | 0.8894 |
| FLAIR | 2, 5, 10 | 0.9113 |
| FLAIR | 5, 8, 9 | 0.8851 |
| T2WI | 1, 4, 9 | 0.9224 |
| T2WI | 1, 6, 8 | 0.9069 |
| T2WI | 2, 4, 7 | 0.9328 |
| T2WI | 2, 7, 8 | 0.9476 |
| T2WI | 7, 8, 9 | 0.9126 |

Question: The answer to this question is incomplete. “The reply to this question is incomplete. Please specify whether you have performed normality testing of the feature distribution to choose Mann-Whitney U test. Also, specify the type of d-values presented.

Now, the modification on p 12, line 288 have given more concerns regarding the statistical testing. Mann-Whitney U test is performed to assess if two distribution as significantly different. However, since the authors claim that the prediction values were converted to binary as correct or wrong, then this statistical testing cannot be applied.”

RESPONSE:

Thank you for pointing this out. I apologize for my lack of knowledge about statistics. The Shapiro–Wilk test showed that the data did not follow a normal distribution (p<0.000); therefore, Mann-Whitney U test was performed. (p.13, line 322)

A cross tabulation was performed to compare the number of correct and incorrect numbers for AI and Dr. Results have shown that AI is superior in diagnosis, and we believe that we have demonstrated the effectiveness of AI. (p.13, line 325)

Thank you again for giving us the opportunity to strengthen our manuscript with your valuable comments and suggestions. We have worked hard to incorporate your feedback, and hope that our revised manuscript is suitable for publication.

Yours sincerely,

Kyoya Sakashita, M.D., Nobuhiro Mikuni, D.D., Ph.D.

Department of Neurosurgery

Sapporo Medical University School of Medicine

S1, W16 Chuo-ku Sapporo

Hokkaido 060-8543, Japan

Tel: +81 11-611-2111

Fax: +81 11-614-1662

Email: kyoya.s628@gmail.com
